# Diseases of the Nasopharynx in Infancy1Read before the Buffalo Academy of Medicine, February 12, 1907.

**Published:** 1907-05

**Authors:** John Lovett Morse

**Affiliations:** Assistant Professor of Pediatrics, Harvard Medical School; Assistant Physician at the Children’s Hospital and at the Infants’ Hospital, Boston


### Diseases of the Nasopharynx in Infancy.[1]

BY JOHN LOVETT MORSE, A. M., M. D.
Assistant Professor of Pediatrics, Harvard Medical School; Assistant Physician at the Children's Hospital and at the Infants' Hospital, Boston.

MY experience leads me to believe that the importance of the diseases of the nasopharynx in infancy and the frequency of their occurrence are not appreciated by the general practitioner, and that they are often either entirely overlooked or insufficiently and improperly treated. It would be impossible for me, even if I desired to do so, to take up all of these diseases, even cursorily, in the time at my disposal; consequently I shall speak only of those with which I am most familiar and which seem to me to be the most important. I shall consider them, moreover, entirely from the point of view of the pediatrician and general practitioner, which is the only way in which I am competent to consider them, and not from that of the nose, throat, and ear specialist. Before speaking of the diseases of the nasopharynx in infancy, it will be well to run over the anatomy of this region at this age.

### NOSE.

The nose is relatively small, the respiratory portion being very small. The height of the posterior nares at birth is from 6 to 7 mm. and the breadth between the pterygoid processes at the hard palate 9 mm. The nasal cavity consists of an upper olfactory region occupying the ethmoidal portion of the cavity, and a lower respiratory region occupying the maxillary part. The nasal cavity at birth is relatively long and shallow, the respiratory portion being very narrow. The whole opening of the posterior nares on either side is just large enough to admit the end of a medium-sized male catheter. The nasal cavity begins to increase in height directly after birth, increasing pretty rapidly during the first six months but very slowly during the remainder of infancy. The size of the posterior opening doubles in six months, after which it remains stationary until the end of the second year. At the end of the seventh month the nasal cavity begins to approach the adult shape though it is still relatively broad.

### VOMER.

The posterior edge of the vomer is nearly horizontal at birth and remains very oblique throughout infancy.

1 Read before the Buffalo Academy of Medicine, February 12, 1907.

### NASOPHARYNX.

The nasopharynx is very low at birth but is relatively long from before backward, the distance from the hard palate to the soft parts of the back of the pharynx being nearly as great at birth as in the adult. The nasopharynx at birth is, therefore, merely a narrow passage running obliquely backward and downward from the constricted opening of the posterior nares. The soft palate is placed more horizontally than in the adult. The height of the nasopharynx increases in the same way as that of the posterior nares.

### EUSTACHIAN TUBES.

The Eustachian tube is nearly horizontal at birth but slants a little during the latter part of infancy. The opening at birth is at the level of the hard palate. It remains at this level for nine months, but later becomes distinctly higher. The tube is not only relatively but absolutely wider at its narrowest point at birth and during infancy than in the adult. This may explain the ease with which catarrhal processes travel to the middle ear in infancy. The prominence at the back of the pharyngeal opening of the tube is but slightly developed in infancy and the fossa of Rosenmuller is therefore almost imperceptible. Catheterization of the tube at this age is, therefore, practically impossible.

### EARS.

At birth and during infancy the external auditory canal runs inward and downward and the membrana tympani is almost horizontal. In introducing the aural speculum, therefore, the ear must be drawn forward and downward instead of upward and backward or the canal will be bent on itself. The petrosquamosal suture is open at birth allowing, therefore, a close connection between the blood vessels of the brain and those of the middle ear. It is said to close at one year, although this is somewhat doubtful. The mastoid antrum is present at birth but the mastoid cells very seldom develop during infancy. The maxillary sinus is well developed at birth. The frontal sinuses are not developed either at birth or during infancy.

### PHARYNX.

The connective tissue between the pharynx and the spine is very lax. The nasopharynx is extremely vascular and there is an abundant supply of lymphatic glands and vessels, especially in the posterior wall. The retropharyngeal lymph nodes form a chain on both sides of the median line of the pharynx, extending from its upper portion to its junction with the esophagus. They lie between the prevertebral aponeurosis and the muscles of the pharynx.

The lymphoid ring is well developed at birth and often more so during infancy.

ACUTE RHINITIS.

The first disease of which I shall speak is acute rhinitis, or the common "cold in the head." This, while merely a disagreeable incident in childhood and adult life, is often a serious and sometimes a fatal malady in infancy, especially in the early months. Owing to the anatomical peculiarities already mentioned a comparatively slight swelling of the nasal mucous membrane completely closes the nose and entirely prevents nasal respiration. This, of course, necessitates oral respiration which the young infant performs very imperfectly, especially when asleep. In fact, sleep is so broken in many instances that the baby gets little if any rest and on this account loses strength very rapidly. The occlusion of the nares also prevents proper sucking and interferes a great deal with swallowing, even when the food is given with a spoon or dropper. Consequently the infant takes but little food or refuses it entirely, even if very hungry. The temperature in these cases is always elevated, usually irregular and very often high. Loss of weight and strength is very rapid as the result of the insufficient supply of fresh air, lack of sleep and deprivation of food, and in feeble babies may be so great as to cause death.

The following is an example of a moderately severe case of acute rhinitis in an infant of three months, that was in poor general condition as the result of an acute attack of gastric indigestion. She began to snuffle during the night of January 23 and slept poorly. Her temperature was 101°F. on the morning of January 24 but rose to 103.4°F. during the day, the pulse going to 160 and the respiration to 70. Nasal respiration was completely obstructed and she lay with her mouth open and her tongue out. She was able to swallow but took almost no food and slept scarcely at all. There was no improvement during the night of the 24th. The next morning the temperature was still high, while the pulse had risen to 180 and the respiration to 80. She was very weak and slightly cyanotic. In short, her condition was such as to justify great anxiety. Fortunately, improvement began at this time and continued to recovery.

The treatment of acute rhinitis may be divided into local and general, the latter being merely sustaining in character. Internal medication in my experience is of little use, although I think that tincture of euphrasia, in doses of two or three drops every hour, is of some benefit in the early stage of watery secretion. Liquid albolene or albolene combined with menthol and camphor in the proportion of one grain of each to the ounce is the most useful local application. It is best introduced with a dropper, the infant lying on its back. In more severe cases a 1-5000 solution of adrenalin chloride introduced in the same way helps a good deal. It is rarely if ever justifiable to use cocaine at this age. In the severest cases it may be necessary to introduce a small rubber catheter into each nostril in order to keep the passage open and to enable the infant to breathe. I am sure that in one of my cases this procedure saved the baby's life.

The general treatment consists of fresh air, food and stimulation. Pure fresh air not only does not do a rhinitis any harm but does it much good, the general superstition to the contrary. If the infant cannot suck it must be fed with a spoon or dropper. If it does not take enough in this way it must be fed through a stomach tube. Strychnia and brandy are the best stimulants.

A complicating inflammation of the middle ear is very common in acute rhinitis because of the anatomical peculiarities already mentioned. Suppurative inflammation of the maxillary antrum almost never develops. It is impossible to tell, however, how common milder grades of inflammation may be. The frontal sinuses not being developed are, of course, not involved.

Acute rhinitis is, in my opinion, a contagious disease, communicable from one person to another. Since it may be such a serious and dangerous disease in infancy, babies should be kept away from adults and older children suffering from "colds" almost as carefully as from those with the eruptive diseases. Fresh air, either in the house or out of doors, does not cause rhinitis but is, next to the avoidance of exposure to other cases, the best preventive. A draft is hardly more dangerous. Babies that get plenty of fresh air both day and night and who are accustomed to go out in all sorts of weather have fewer "colds" than any others and bear them better when they do have them.

### DIPHTHERITIC RHINITIS.

Diphtheritic rhinitis (membranous rhinitis) is much more common in infancy than is usually supposed. It is generally of a very mild type and is usually not suspected until other children come down with pharyngeal or laryngeal diphtheria, and often not even then. It is, perhaps, the most common source of the epidemics of diphtheria which develop in hospitals and institutions for infants and children. My experience at the Infants' Hospital has taught me to suspect every nasal discharge in an infant, especially if it is irritating. In spite of the greatest care in the admission of cases we found it impossible to avoid epidemics of diphtheria from this cause until routine immunisation was adopted. The babies now receive 500 units of antitoxin on admission and every three weeks thereafter. Since this practice was begun six years ago but one case of pharyngeal diphtheria has developed in the patients in the hospital, although many unimmunised nurses and nursery maids have come down with it and many cases of diphtheria have been discovered.

The symptoms of nasal diphtheria in infancy are, as a rule, very mild. There is usually a little snuffles with a slight nasal discharge and a little nasal obstruction. There is no glandular enlargement and the constitutional symptoms are wanting or very mild. The nasal discharge, however, is often rather characteristic, being thin, watery and acrid but, contrary to the usual statements, rarely bloody. It frequently irritates the upper lip, causing crusts about the nares and on the lip. A thin, watery, nasal discharge which irritates the lip should in an infant always be regarded with great suspicion. Such discharges should always be examined bacteriologically. The amount of the discharge varies markedly in different cases. There is almost never any membrane visible unless the speculum is used, and even then it may not be seen. The diphtheritic process practically never spreads backward and downward to the pharynx and larynx. The diagnosis must depend, therefore, in most cases on the bacteriological examination of the nasal discharge.

These cases must be isolated in the same way as cases of pharyngeal and laryngeal diphtheria. They should be given antitoxin freely. Alkaline washes introduced with a dropper, or in more severe cases with a syringe, are of some use. Strong antiseptic solutions should not be used.

In some instances, however, even in infancy, nasal diphtheria takes a more severe form. Even in these cases, however, the symptoms are due largely to nasal obstruction and not to toxemia and resemble very closely those of acute rhinitis. One of my cases in an infant of three months died in four days, apparently solely as the result of nasal obstruction and not of toxemia. No membrane was seen either by myself or by a throat specialist on several examinations. The true condition was not suspected and no bacteriological examination was made. The diagnosis of nasal diphtheria seems justified, however, from the fact that two or three days later the mother developed a very severe case of pharyngeal diphtheria.

### ADENOIDS.

While the frequency and importance of adenoids in childhood is well recognised and their symptomatology pretty generally understood, this is not the case in regard to adenoids in infancy.

They are supposed to be uncommon and of little importance, and their symptoms are very commonly overlooked or misinterpreted. They are, however, very common in infancy and of great importance. Their symptomatology, while in many ways different from that in childhood, is nevertheless, fairly characteristic. The evil results which they produce are, moreover, even greater in infancy than in childhood. There is, unfortunately, a general feeling in the profession, even among the throat specialists, that adenoids should not be removed in infancy because they are liable to recur later. For this entirely insufficient reason the unfortunate babies are allowed to suffer, to have their development seriously or irreparably interfered with, and even to die. The fact is that, in the majority of instances, the adenoids do not recur, but even if they do and a second operation is required, that is no reason why the poor babies should be subjected during the interval to all the evils which adenoids produce, when they might be avoided by an early operation. The removal of adenoids in infancy is not a difficult operation and is practically devoid of danger. Certainly there is no danger connected with the operation which should be considered for an instant, in comparison with the evil and even dangerous results which adenoids may and often do produce at this age.

On account of the small size of the superior pharynx and post-nasal opening, even a small amount of adenoids may cause marked obstruction to nasal respiration. The seriousness of any interference with nasal respiration in the infant has already been noted in speaking of acute rhinitis. The constant deprivation of a sufficient supply of oxygen produces a condition of malnutrition which no method of feeding or mode of life influences in any way. No improvement can take place until the adenoids are removed or nature partially relieves the obstruction by the growth of the parts. Rickets is especially prone to develop in these cases and considerable deformities of the chest are sometimes produced. These are unimportant, however, compared with the general disturbance of nutrition. Another very serious result of the nasal obstruction is the interference with sucking. It is such an effort to suck that these babies take only enough food to satisfy the acute pangs of hunger. Lack of food, therefore, also interferes with their nutrition and development. The difficulty in nasal respiration makes them restless at night and interrupts their sleep, which again disturbs their nutrition. It is surprising how quickly and markedly they will improve in general condition after the adenoids are removed.

The following case shows most strikingly to what extent adenoids may disturb the nutrition in infancy and how great the benefit may be from their removal. This baby began to have snuffles when he was about three months old and soon after to have a little difficulty in breathing, especially at night. He took his food fairly well but ceased to gain when he was 4½ months old. He managed to sleep pretty well. His head began to sweat freely at 4½ months and a week or two later the mother noticed that his head was "soft." About the same time he began to have frequent attacks of laryngismus stridulus, especially when taking his bottle. At five months he began to have convulsions. He was seen when six months old. He was then rather poorly developed and nourished. He was moderately pale and there were circles about his eyes. His mouth was open and but little air passed through his nose. There were marked craniotabes and a moderate rosary. The throat was normal to inspection but adenoids were felt with the finger. These were removed a few days later. He began to improve almost immediately. He had no convulsions or attacks of laryngismus after the removal of the adenoids. He took his food better and soon began to gain in weight. Two months later he was in splendid condition, the head solid, the color good, the mouth shut and the breathing quiet.

Adenoids are one of the commonest, if not the most common, cause of chronic "snuffles" in infancy. They are also almost always present in those babies that are subject to frequent "colds in the head," and are of great etiological importance in this connection. They are usually overlooked, however, because the baby does not keep its mouth open, snore at night or have the typical facies of adenoids in later childhood, there being apparently a very general impression that there can be no adenoids, or at any rate no adenoids of importance, unless these symptoms are present. This is not so, of course, frequent colds and chronic snuffles being almost as suggestive and characteristic of adenoids in infancy as these more marked symptoms are in childhood. The treatment of these affections is most unsatisfactory, moreover, unless the underlying condition is recognised. When this is done and the adenoids removed, the "colds" and "snuffles" cease almost immediately. Operation is not always necessary, however, as mild cases often yield to local astringent and stimulating treatment. I have found the following mixture very useful

| | | |
|---|---|---|
| ℞ | Iodine | gr. ¼ to gr. ½ |
| | Camphor | gr. 1 |
| | Menthol | gr. 1 |
| | Benzoinol | oz. 1 |

Five to ten drops of this mixture are put into the nose with a dropper every three or four hours. This, by the way, is the best method of making applications to the nose and nasopharynx in infancy. Sprays are of little use at this age because of the fright and struggling which they induce. The drops are best put in with the baby lying on its back so that they may run through into the nasopharynx. Although these cases are so common and the relief from proper treatment, especially from operation, is so striking, it hardly seems necessary to give illustrative cases, as the condition is easily recognised if it is only borne in mind.

Adenoids not only cause "snuffles" and "colds" but also very frequently an irritating cough without physical signs. This cough is especially troublesome at night. Here again, treatment, unless the real source of the trouble is recognised, is of no avail. Cough mixtures and throat sprays are useless. The cough can only be controlled by stupefying the infant with bromide or paregoric. Treatment of the adenoids or their removal, however, is almost immediately successful. The following case is an example.

A baby, 5½ months old, that had never gained very well and had taken her food poorly, began to have snuffles which were soon followed by a cough at night which kept her from sleeping. The throat and lungs showed nothing abnormal. Nasal respiration was fairly free and the discharge from the nose was insignificant. The adenoids were removed when she was seven months old. The cough ceased after 24 hours and never recurred. Improvement in her general condition also began at once and continued.

Adenoids are, either directly or through the colds which they induce, undoubtedly the most common cause of otitis media in infancy. Repeated attacks of otitis media are almost invariably due to adenoids and the attacks continue to recur until the adenoids are removed. The symptomatology of otitis media in infancy will be referred to later.

Adenoids are also a very common cause of sleeplessness and restlessness at night in infancy, even when there are no marked symptoms like mouth breathing, snuffles or cough. They should always be thought of when babies sleep poorly.

The development of adenoids opens the doors of the lymphatic ring of the pharynx still wider for the entrance of bacteria and their toxic products. They are, therefore, often accompanied by and are undoubtedly frequently the cause of enlargement of the cervical lymph nodes in infancy. These nodes are not infrequently tubercular. Tuberculosis of the middle ear may also be secondary to adenoids, whether they are or are not themselves tubercular. That tubercle bacilli may be present in and pass through adenoids at this age is shown by one of my cases in which, through a mistake in diagnosis, the adenoids were removed from an infant of 5 months during the early stage of tubercular meningitis, tubercle bacilli being found in the adenoid tissue. The danger of tubercular infection from adenoids is, therefore, a real one and furnishes another reason for their removal.

PHARYNGITIS.

There is another condition in infancy which I have seen very often and which I have found very difficult to treat satisfactorily, but about which I hesitate to speak because I have never heard anyone else mention it, presumably because they have not thought it worth while. For lack of a better term I am in the habit of calling it "acute pharyngitis." The pharynx and fauces are usually only moderately reddened, but considerably swollen and markedly edematous. The condition never goes on to abscess formation. It is usually associated with a slight nasal irritation and snuffles. Cough is very troublesome especially during sleep, which is usually much disturbed. Paroxysms of difficult breathing, at times amounting to orthopnea, sometimes occur at night. Sucking and deglutition are interfered with and but little food is taken. The larynx is never seriously involved but a secondary bronchitis is not uncommon. The temperature is always elevated, usually moderately, sometimes considerably. The usual course is from a few days to a week, but it sometimes drags on for several weeks. Recovery is the rule but death sometimes occurs, apparently from exhaustion. Treatment is unsatisfactory. The iodine mixture, already spoken of, in the nose, is of some benefit. Glycerite of tannin applied locally with a swab has seemed to do more good than anything else. Drugs internally have proved useless. The chief reliance must be placed, however, on feeding, with the tube if necessary, and stimulation.

RETROPHARYNGEAL ABSCESS.

The retropharyngeal lymph nodes drain the cavities of the cranium, pharynx, nose and middle ear. They are, therefore, liable to infection from any of these cavities. Infection results, as with other lymph nodes, in simple adenitis or suppuration. Several nodes are usually involved in simple adenitis, but suppuration rarely takes place in more than one. Suppurative inflammation is accompanied by a cellulitis of the surrounding tissues. Owing to the looseness of the connective tissue between the pharynx and the spine large accumulations of pus are possible. Any of the pus-forming organisms may cause suppuration and the tubercle bacillus has been found in a few cases. Retropharyngeal adenitis always precedes retropharyngeal abscess. The symptoms of retropharyngeal adenitis are, however, not characteristic, being very similar to those of a pharyngitis. Inspection shows nothing definite but the enlarged nodes can usually be easily felt by the finger. Adenitis usually results in resolution but occasionally goes on to suppuration. Originating, as it always does, in a single laterally placed node, the abscess is situated to one side of the median line and is in the lateral wall rather than in the back of the pharynx. It may be high up or low down according to the location of the node which is involved.

The first symptom of retropharyngeal abscess is usually unwillingness to take food or difficulty in swallowing. In the beginning this is probably due to pain, later to the mechanical obstruction of the tumor. Modification of the voice and difficulty with respiration next develop. These vary according to the location of the tumor. If the abscess is in the upper portion of the pharynx the cry is nasal and nasal respiration is chiefly disturbed. The mouth is kept open and the breathing is snoring and snuffling. If the abscess is low down the cry is laryngeal and the respiration is stridulous, like that in laryngeal spasm or stenosis. In these cases the respiratory murmur is diminished and many moist rales are heard. These may be due to a complicating bronchitis but are usually the result of the interference with respiration. In the acute cases the head is held in a most characteristic position from the first, the neck being extended and the head turned to one side. In the subacute cases this may not apear for some time. Swelling of the lymph nodes at the angle of the jaw on the affected side soon develops and may become very marked. The temperature is usually high and irregular.

The following case in an infant of 19 months, which developed under my observation, is fairly characteristic, although the swelling in the external lymph nodes appeared earlier than is usual. It was noted Nov. 10 that he was feverish, did not care to eat and kept putting his hands in his mouth as if it was sore. Careful physical examination, including inspection of the mouth and throat, showed nothing abnormal except slight enlargement of the lymph nodes about both sternomastoids. The temperature was 103°F., the pulse 160 and the respiration 56. The next day he held his head rather rigidly and cried if it was moved. The cervical lymph nodes were rather more swollen, especially on the left. The throat was still negative to inspection. The next day the head was held very rigidly and bent toward the left shoulder. The swelling of the lymph nodes was larger but the throat was negative both to inspection and palpation. The temperature ranged between 101°F. and 104°F. On the 13th he began to have a good deal of mucus in his throat, and digital examination showed a little indefinite soft swelling in the left side of the pharynx. The condition was otherwise unchanged. On Nov. 14 there was much difficulty in swallowing. The swelling of the cervical lymph nodes and the position of the neck remained unchanged. The swelling in the pharynx gradually increased but did not become distinctly elastic until the 18th, when it was opened and a teaspoonful of pus let out. The opening closed during the night and the swelling was much larger on the nineteenth. It was then opened very freely and two teaspoonfuls of pus let out. He slept all the next night and began to take more food almost immediately. The abscess continued to discharge for 3 or 4 days longer and the swelling was entirely gone on November 26.

This case was operated on as soon as enough pus had formed to show where to make the incision, and none of the severe symptoms of retropharyngeal abscess had time to develop. The following case, seen in consultation, in which the condition was unrecognised and untreated, illustrates the symptomatology of neglected cases.

Five weeks previously an enlarged lymph node was noticed in the right side of the neck of a nine months old boy. This node gradually increased in size and others appeared. At the same time he began to take his food rather poorly, as if swallowing was difficult and painful. Finally he began to have some little trouble in breathing. The difficulty in swallowing and in breathing had steadily increased, so that during the last 48 hours he had been unable to swallow at all. When he lay down he immediately choked up so that he had to be held in a sitting position. At times the difficulty with respiration became so great, even when sitting up, that it was feared that he would suffocate. He had taken no food by the mouth for 36 hours. Rectal feeding had been attempted but the enemata were not retained. He had lost some weight and much color. The temperature had been moderate. There had not been much cough. There was no cyanosis when he was quiet, but when he cried it was marked. He could not lie down at all but was fairly comfortable sitting up. His mouth was wide open; the respiration, however, was not noisy. There was slight retraction of the epigastrium and supraclavicular spaces even when he was quiet. There was marked swelling of the lymph nodes on the right side of the neck, but no involvement of the surrounding tissues, fluctuation, redness or heat. The whole right side of the throat was filled up with a large red mass. The tonsil and soft palate were pushed well forward. Palpation showed that this mass reached high up into the nasopharynx, filled up the whole right side of the pharynx and extended downward over the larynx, leaving merely a chink through which the air entered. The larynx itself was normal, as were the heart and lungs.

The abscess was opened by a long, deep incision, the finger being used as a guide. Nearly two tablespoonfuls of dirty green pus were obtained. Relief was almost immediate. He began to breathe at once with his mouth shut, lay down and went to sleep. A few hours later he began to take food. Recovery was rapid and complete.

Although the symptoms of retropharyngeal abscess are usually so characteristic, they are very frequently misinterpreted.

If the obstruction is high up, they are most often mistaken for those of a simple pharyngitis or rhinitis, although diphtheritic paralysis is sometimes suspected. If the obstruction is low down they are often ascribed to laryngitis, either catarrhal or diphtheritic. In others, in which the signs of bronchitis are marked, the primary condition in the throat is entirely overlooked and the diagnosis of bronchitis or broncho-pneumonia made.

The chief reason that a retropharyngeal abscess is so often overlooked is that it is not thought of, and hence is not even considered in the differential diagnosis. Another reason is that inspection of the throat is trusted and palpation omitted. Inspection, however, is often insufficient and may lead to grave errors in diagnosis. In the majority of cases the tumor is visible, but not infrequently, especially if low down, it cannot be made out. It can always be felt if present, although if it is situated low down the finger must be introduced very deeply. In no case in which the symptoms in any way suggest a retropharyngeal abscess should palpation of the pharynx be omitted. In fact, it should always be done in every case in which there are symptoms of respiratory obstruction and also in all cases in which the symptoms point to the respiratory tract but in which the physical signs do not satisfactorily account for the symptoms. It causes the infant only temporary discomfort and may prevent very serious mistakes in diagnosis. The swelling is usually situated in the side of the pharynx but may be at any level. It is either tense and elastic or fluctuating. The examination of the throat should be made with a tongue depressor and not with a gag, as immediate death has in several instances followed the introduction of a gag. Death under these circumstances is probably due to some disturbance of the vagus from the pressure of the abscess.

A sudden attack of dyspnea may sometimes be the first symptom to call attention to the local condition in the throat. In feeble infants a small abscess may develop so insidiously as to cause neither general nor local symptoms.

The prognosis of retropharyngeal abscess depends largely on the treatment. The usual termination in untreated cases is in death. This may occur slowly as the result of progressive weakness or asphyxia, but more often suddenly from laryngeal spasm or some disturbance of the function of the pneumogastric nerve. The abscess seldom opens spontaneously. If it does, and the opening is into the pharynx, death may result from suffocation or from a consecutive inspiration pneumonia. The pus may burrow in various directions, sometimes opening externally and occasionally into the middle ear or external auditory canal. In a few instances the carotid artery has been opened. The prognosis in cases which are properly treated, that is, by incision, is very different, however, the mortality in such cases being only about five per cent.

The true condition is seldom recognised early enough to have cold applications externally do any good. It is much more reasonable to endeavor to hasten suppuration by the use of poultices and hot applications to the throat. Spontaneous evacuation should never be awaited. The abscess should be opened as soon as the evidences of suppuration are present. It does no harm to make an incision even before this happens. Incision through the mouth is much preferable to incision through the neck. The drainage from the internal incision is almost always satisfactory and, if not, can be made so by another incision or by dilating the original one. Secondary infection through the wound, while possible, is so unusual that it ought not to be considered in any way to contraindicate the internal incision. The external operation is a delicate one and requires a skilful surgeon and assistants, while the internal operation can be performed by any physician at any time without skilled aid. The external operation, moreover, requires dressing and leaves a scar, while the internal calls for nothing but cleansing of the mouth and the occasional expression of the contents of the abscess with the finger.

The incision is best performed with the infant in the upright position. If it is tipped forward the instant the incision is made there is no danger of the pus entering the air passages. The knife should be guarded except at the point. A gag should never be employed because of the danger of sudden death from its use, but the mouth held open with the finger or a tongue depressor. With these also care must be taken not to open the mouth too widely. It is usually well to enlarge the incision with the finger in order to favor thorough drainage. The abscess should be squeezed once or twice a day with the finger in order to keep up drainage and to prevent the opening from closing. The mouth should be washed out from time to time with some mild antiseptic solution.

### OTITIS MEDIA.

While, strictly speaking, otitis media is not a disease of the nasopharynx yet it is in infancy so almost invariably secondary to diseases of the nose and nasopharynx that it seems justifiable to speak of it in this connection. The anatomical peculiarities of the nose, nasopharynx and Eustachian tubes in infancy, already referred to, explain the frequency of otitis media at this age. In some cases the catarrhal inflammation undoubtedly extends directly up the Eustachian tube, while in others the primary lesion is probably a blocking of the tube with a secondary inflammation. There is no condition in infancy, I think, regarding which there is a more general lack of knowledge among the profession as a whole and a more general misapprehension as to the symptomatology than otitis media. Very few physicians seem to realize the frequency of this condition in infancy and its importance in differential diagnosis. I have learned to look for it in all cases of doubtful diagnosis and to consider it as a possible cause for any symptoms not otherwise easily explainable in the course of other diseases. No physical examination in infancy, in which other definite signs sufficient to account for the symptoms are not found, should be considered complete without an examination of the ears. They should be considered especially when there have been, or are, diseased conditions of the nose or nasopharynx present, even if these are very slight.

One reason why otitis media is so frequently overlooked is, as already stated, the general misapprehension as to the symptomatology of the disease at this age. Physicians are constantly on the lookout for earache and tenderness over the mastoid and, if they do not find them, rule out trouble in the ear without further investigation. On the other hand, they rarely think of inflammation of the ears as the possible explanation of an obscure fever or of peculiar symptoms without obvious cause. Many infants with acute inflammation of the middle ear, however, show no signs of pain at any time, and many others, who evidently have pain somewhere, show nothing by their actions which will attract the attention of the physician to the ears. Babies with earache rarely put their hands to their ears. On the other hand, sick babies are very likely to wave their arms about, to grab at their ears and pull their hair. The usual symptoms of earache are sharp cries, restlessness and inability to sleep. Apparent tenderness on pressure over the mastoid is another most unreliable guide. Tenderness over the mastoid is very unusual in simple acute inflammation of the middle ear, and, on the other hand, most sick babies will cry out if firm pressure is made on any part of their body. Mastoid inflammation is very rare at this age on account of the undeveloped condition of the mastoid cells. Tenderness over the mastoid can, therefore, hardly be expected. In fact, in most of the cases of otitis media in infancy there are no symptoms which attract attention directly to the middle ear as the cause of the trouble. Acute inflammation of the middle ear is usually, but not always, associated with fever, which is often very high. It is also very commonly associated with reflex nervous symptoms which direct attention away from the ears to other parts of the body. Among the more common of these reflex symptoms are rapid respiration, vomiting and tenderness and rigidity of the neck. Convulsions are not very infrequent. On account of these reflex symptoms almost every disease but the right one is suspected, among which may be mentioned meningitis, pneumonia, gastritis, worms and dentition.

The following is a fair example of the ordinary case of acute otitis media, there being nothing about the symptoms to direct the attention to the ear except a slight "cold in the head." An infant, 13 months old, had had a slight "cold in the head" with loss of appetite for several days but no fever until the day before. During the previous night she had been hot and restless and in the morning was somewhat drowsy and stupid. She coughed a little as if her throat was sore and refused her food. Careful examination showed nothing abnormal except slight enlargement and reddening of the tonsils. The temperature was 103°F., the pulse 160 and the respiration 60. Further examination showed reddening and bulging of both ear drums. The ears were opened in the afternoon with a free discharge of pus from both. The temperature dropped to 99°F. and the pulse to 120 during the next twelve hours.

The following cases show how marked and how confusing the reflex symptoms of otitis media may be and how easily they may draw attention away from the real seat of trouble. A six months old baby was taken suddenly sick Feb. 18th with fever, cough and difficulty in breathing. He lost his appetite but showed no other symptoms of gastroenteric disturbance. He was taken to a physician Feb. 21st who found the temperature 104.2°F., the pulse 160 and the respiration 52. He sent the baby to the Infants' Hospital with the diagnosis of pneumonia. The physical examination was not made until the next day. The temperature was then 100°F., the pulse 115 and the respiration 38. The physical examination was entirely negative and while the picture was not that of a pneumonia and the lungs were negative, the disproportion between the rate of the pulse and that of the respiration pointed to a certain extent to some pulmonary trouble. That night the temperature and pulse rose rapidly while the rate of the respiration remained the same, thus pointing much less strongly to the lungs. The next day, the 23d, the ears were examined and considerable reddening of the right membrana tympani was found. Bulging occurred the next day. Paracentesis was performed and a small amount of pus let out. The left drum was also reddened but did not bulge. The temperature immediately dropped and showed a tendency to run lower. On the 26th, however, it rose again, as did the pulse and respiration, the respiration rising again out of proportion to the pulse. Nothing could be found in the lungs, however. Examination of the ears showed that the right ear was not discharging freely and that the left drum was bulging. Paracentesis was done on the left ear and the discharge re-established in the right. The temperature and pulse dropped immediately and were followed in 24 hours by the respiration, all remaining normal afterward. In this case the onset and general picture in the beginning was those of pneumonia and although nothing abnormal could be found in the lungs, the disproportion between the pulse and the respiration continued to point strongly toward them. The diagnosis would have remained obscure without the examination of the ears

A baby, 6½ months old, was very fussy for 24 hours, was constantly putting her hands in her mouth, cried if her hand was taken out of her mouth and acted as if her mouth was painful. The temperature was 105.2°F. The physical examination, which did not include the ears, showed nothing abnormal except a slight swelling of the gum over the lower middle incisors. A diagnosis of difficult dentition was made and the gum lanced over the lower middle incisors. Lancing the gums was followed by temporary relief, but within 24 hours the temperature was up again and all the symptoms had recurred. The ears were then examined and reddening and bulging of the left drum found. Paracentesis was followed by a profuse sero-sanguinolent discharge with marked relief of the symptoms and a rapid fall in the temperature, which did not rise again. In this case it would have been very easy to have continued to attribute all the symptoms to difficult dentition, as was done when the infant was first seen.

In the following case the symptoms were most unusual and misleading, all pointing to some obscure cerebral condition, either acute or chronic. The true condition was not suspected, although the patient was seen by a number of men, all of whom were well aware of the importance of ear disease in infancy as well as of the variety of its symptomatology.

A baby, aged 15 months, had a good family history. There was no history of nervous or mental disease or anything to suggest syphilis. She was delivered by instruments, the head presenting, and was normal at birth. She had always been well except for chicken-pox at 13 mos. Her parents said that she was unusually bright mentally. She had walked alone for a month or more.

On Dec 13 she tripped on the floor and fell, striking her head on a chair. She did not appear to be much hurt. Dec. 22d she began to have "convulsions" which continued up to the time of entrance to the Infants' Hospital, Dec. 30. She had a slight cold and vomited several times daily. The bowels moved daily and the movements were normal. She was in good general condition but there was moderate pallor of the skin and mucous membranes. There were no evidences of rickets. The fontanelle was 1 ½ cm. in diameter and slightly depressed. She did not open the left eye quite as wide as the right but closed both vigorously. The motions of the eyes were normal and the pupils reacted quickly to light. She both saw and heard. There was no rigidity of the neck or back. There was no spasm or paralysis of the extremities. The knee-jerks were equal and somewhat exaggerated. Kernig's sign was absent. All the organs were normal. The urine was pale, acid, of a specific gravity of 1008 and contained no albumin.

She slept well and was perfectly quiet when asleep. When awake there was almost always tremor of the head and arms, although she was sometimes quiet for a short time. This tremor was rhythmic and very rapid. It was most marked in the head and arms, although it was also present in the body and legs. She was very easily excited and frightened. She then became rigid and threw herself about, crying loudly. The cry was interrupted at short intervals but the rhythm was much less rapid than that of the tremor. In these attacks the tremor was much more marked and more rapid. In some of the attacks there was also nystagmus, usually lateral, but sometimes vertical. The temperature was normal at entrance and never went above 99.6°F. The pulse and respiration were somewhat rapid for the first two days, but afterward showed nothing worthy of note.

Her stomach was washed out and her bowels moved with castor oil. The vomiting ceased at once and did not recur. The movements were normal. Moderate doses of bromide and chloral had no effect. Her condition continued unchanged and there was no change in the physical signs. The fundi of the eyes showed nothing abnormal.

She was seen by three specialists in nervous diseases, all of whom agreed that the fall was a coincidence and that there was no acute cerebral process. They said that the history of normal mental development must be wrong and that only a congenital cerebral defect could account for the symptoms. The medical men who saw her did not agree with this diagnosis but also ruled out meningitis and injury as the cause of the symptoms. They were, however, unable to make any diagnosis.

Her ears were examined Jan. 4th as part of a routine examination of all the babies in the hospital. Both drum membranes were generally reddened and bulged in their upper posterior portions. Paracentesis showed pus on both sides. Improvement was almost immediate and after the first few days was very rapid. The ears were normal on Jan. 14th and she was discharged well a few days later. There has been no return of the symptoms.

In the following case the location of the reflex symptoms led to a diagnosis of cervical Pott's disease and later to one of meningitis.

This baby, 16 months old, had a good family history, had always been well and had never been exposed to tuberculosis. She began to be fretful and irritable August 9. She did not move about as much as usual and preferred to lie as quiet as possible. She cried out when moved and also many times in the night. Her appetite became poor and she lost weight steadily. There was a slight diarrhea at first, but after a week the movements became normal in number and character. August 21st she began to hold her head backward and the neck was rigid and tender. She was sent to the Children's Hospital Aug. 22 with the diagnosis of cervical Pott's disease. During the next two days her temperature ranged between 103°F. and 104.6°F, the pulse between 120 and 130 and the respiration between 25 and 30. She continued to hold her head backward and rigidly and movements of the head caused pain. No new symptoms developed. The surgeons ruled out cervical Pott's disease but suspected meningitis.

She was in good general condition. She looked and acted sick but although very fretful was perfectly conscious. The anterior fontanelle was 1 ½ cm. in diameter and level. The pupils were equal and reacted to light. The throat was slightly reddened. All the organs were normal and there were no evidences of rickets. There was no spasm of paralysis of the extremities. Kernig's sign was absent. The knee-jerks were not obtained. She held her head a little backward and motion forward was resisted and seemed to cause a little pain. The cervical lymph nodes were slightly enlarged. The urine was normal. The white blood count was 20,000.

Examination of the ears showed reddening and bulging of both drums. Paracentesis was followed by a free discharge of muco-pus with almost immediate relief of the symptoms.

Since there is so little characteristic about the symptomatology of otitis media in infancy, and since the symptoms are so misleading, this condition should be considered in every acute illness at this age and the ears examined. The only way to determine satisfactorily whether or not there is inflammation of the middle ear is to examine the ear with a speculum. All other methods of examination are incomplete and necessarily misleading. The examination of an infant's ears is not, however, a simple matter and requires considerable experience. The results, however, are well worth the time required to master the technique. The method of introducing the speculum rendered necessary by the anatomical peculiarities of the external auditory canal at this age, has already been referred to. A smaller speculum than comes with most sets is necessary. The infant should be held firmly in the nurse's lap, sitting sidewise, with the head against the nurse's breast. Unless there is plenty of daylight, a kerosene lamp will be found more satisfactory than anything else in examining the ears.

In cases of a more subacute type, the symptoms are mainly those of confined pus, and the hectic temperature, often associated with chills, and the apparent absence of a local cause often lead to the diagnosis of tuberculosis or malaria.

The following case of this variety was mistaken for malaria. A boy, 22 mos. old, who lived in a malarial district, had always been delicate and pale. He had had a cough since an attack of bronchitis three months before and had also had a slightly elevated temperature during this time. During the last two weeks he had seemed worse and the temperature had ranged higher. The night before he was seen he had had a chill, followed by a temperature of 105°F., and sweating. Nothing abnormal had been found on physical examination except pallor and slight enlargement of the spleen. The urine showed nothing abnormal.

An almost positive diagnosis of malaria had been made on the basis of the chills, fever and sweating, the enlargement of the spleen, the pallor and the apparent absence of any cause for the symptoms. Examination of the blood showed no plasmodia and 30,000 white cells, a finding entirely inconsistent with malaria.

An examination of the ears, which had hitherto been overlooked, disclosed the seat of trouble. Pus was found on both sides and evacuated. Improvement was continuous and rapid from that time.

In other cases the inflammation may run its course with little or no rise in temperature and with very slight constitutional, local or reflex symptoms, a discharge from the ear being the first thing noted. This is especially likely to be the case in feeble babies, the amount of reaction apparently depending a good deal on the general condition of the infant.

The following cases are examples. A boy, 3½ months old, had always been improperly fed and had always had colic, vomiting and diarrhea. He had recently lost weight very rapidly. He was admitted to the Infants' Hospital March 12th. He was fairly developed and nourished but rather pale. Physical examination was negative except for a slight rosary. A routine examination of the ears a few days after entrance showed nothing abnormal. He responded very quickly to proper food, stopped vomiting, and after a few days had normal movements but did not gain in weight. On March 26th the temperature suddenly rose to 102.8°F. The pulse and respiration did not rise, however, and no other symptoms developed. Nothing abnormal was found on physical examination until the ears were reached, when both drums were found slightly inflamed. No treatment was instituted. The inflammation in the ears rapidly subsided in the next 24 hours and with it the temperature fell to normal and remained there.

A three months old baby entered the Infants' Hospital March 7th. No history came with her. She was emaciated and the skin was mottled. The anterior fontanelle was small and depressed and there was a slight rosary. The physical examination was in other respects negative. She took her food well, seldom vomited, had nearly normal movements and gained in weight. She had no fever at any time and showed no symptoms pointing to aural trouble. Nevertheless, a discharge from the right ear appeared March sixteenth.

Much has been written as to the importance of the patency of the petrosquamosal suture in infancy, as favoring the extension of inflammation from the middle ear to the meninges. This danger has, in my experience, been very much overestimated, and can, to all intents and purposes, be disregarded. The mastoid antrum is present at birth, but the mastoid cells are almost never developed during infancy. Mastoid inflammation is, therefore, infrequent in connection with inflammation of the middle ear at this age, and if it occurs is usually not as serious as in later life.

The proper treatment of otitis media in infancy requires more knowledge of the ear than is possessed by most general practitioners. It requires special knowledge to determine whether or not a given case requires paracentesis or whether it will quiet down under simple measures. In general it is far wiser in a doubtful case to err by doing an unnecessary paracentesis than by letting it go without operation. Local treatment should be extremely simple: irrigation with warm water, heat externally, bromide internally. Under no conditions should oily substances be introduced into the ear and it is rarely advisable to use any local medication. Paracentesis is not a difficult operation, providing the drum membrane can be distinctly seen. It is almost impossible to do any harm by a paracentesis, if reasonable care is exercised. It is, therefore, a safe operation for the general practitioner. He should be prepared for it and ready to do it whenever necessary.

70 Bay State Road.

---

DIAGNOSIS OF EARLY PREGNANCY WITH REFERENCE TO A PARTICULAR SIGN.—Louis J. Ladinski (*Medical Record,* April 13, 1907,) claims that it is always possible to make a positive diagnosis of pregnancy as early as the fifth or sixth week when intrauterine. The diagnosis is made by a single sign. This is the appearance of a spot of a peculiar softness and elasticity just above the junction of the body and cervix in the anterior wall of the uterus, in the fifth or sixth week. When the uterus is retroverted or retroflexed it appears in the posterior wall, and at the sixth or seventh week of pregnancy. In incomplete abortion or subinvolution this area of softening appears, but is much more doughy and less elastic. With a hard fibroid in the upper portion of the uterus there is a similar spot, but the softness is only relative. With a soft myoma in the front of the uterus the feeling is exactly that of pregnancy. The sign must be sought by bimanual palpation. Absence of this sign absolutely excludes uterine pregnancy. The sign aids in making a positive diagnosis of extrauterine pregnancy by excluding uterine pregnancy. It is in all probability due to extreme vascularity of the uterine wall at the point where it is felt.

